# Validation of the RBP‐9000c Oscillometric Blood Pressure Monitor in the General Population According to the Association for the Advancement of Medical Instrumentation/European Society of Hypertension/ International Organization for Standardization Universal Standard

**DOI:** 10.1111/jch.70194

**Published:** 2025-12-23

**Authors:** Shijie Yang, Zhanyang Zhou, Huanhuan Miao, Yuqing Zhang

**Affiliations:** ^1^ AnZhen Hospital of the Capital University of Medical Sciences Beijing China; ^2^ Department of Cardiology Fu Wai Hospital Chinese Academy of Medical Sciences and Peking Union Medical College Beijing China

**Keywords:** accuracy, blood pressure monitoring, ISO protocol, validation

## Abstract

The aim of this study was to evaluate the accuracy of the single upper‐arm cuff oscillometric blood pressure (BP) monitor RBP‐9000 c developed for office and home blood pressure measurement in the general population according to the Association for the Advancement of Medical Instrumentation/European Society of Hypertension/International Organization for Standardization (AAMI/ESH/ISO) Universal Standard (ISO 81060–2:2018). Subjects were recruited to fulfill the age, gender, BP, and cuff distribution criteria of the AAMI/ESH/ISO Universal Standard in the general population using the same‐arm sequential BP measurement method. The test device incorporates a single built‐in cuff suitable for 17–42 cm arm circumference. For validation criterion 1, the mean ± SD of the differences between the test device and reference BP readings was 2.4 ± 6.7/3.3 ± 6.3 mmHg (systolic/ diastolic). For criterion 2, the SD of the mean BP differences between the test device and reference BP per subject was 5.28/5.32 mmHg (systolic/diastolic). The RBP‐9000c oscillometric device for office and home BP measurement fulfilled all the requirements of the AAMI/ESH/ISO Universal Standard (ISO 81060–2:2018) in the general population and can be recommended for clinical and self‐use at home.

**Trial Registration**: ChiCTR2300075747

## 1 Introduction

The accurate measurement of blood pressure (BP) is an important prerequisite for the reliable diagnosis and efficient management of hypertension and other medical conditions. Therefore, the evaluation of the accuracy of automated devices available on the market for BP measurement in the medical environment and the community is of paramount importance [1]. This validation study assessed the blood pressure (BP) measurement accuracy of the new oscillometric upper‐arm cuff device RBP‐9000c (Shenzhen Raycome Health Technology Co, Ltd) developed by researchers from the FuWai Hospital, Chinese Academy of Medical Sciences and Peking Union Medical College for office and home BP measurement according to the Association for the Advancement of Medical Instrumentation/European Society of Hypertension/International Organization for Standardization (AAMI/ESH/ISO) Universal Standard (ISO 81060–2:2018) in the general population [1, 2, 3].

## 2 Methods

### 2.1 Test Device

**FIGURE 1:**
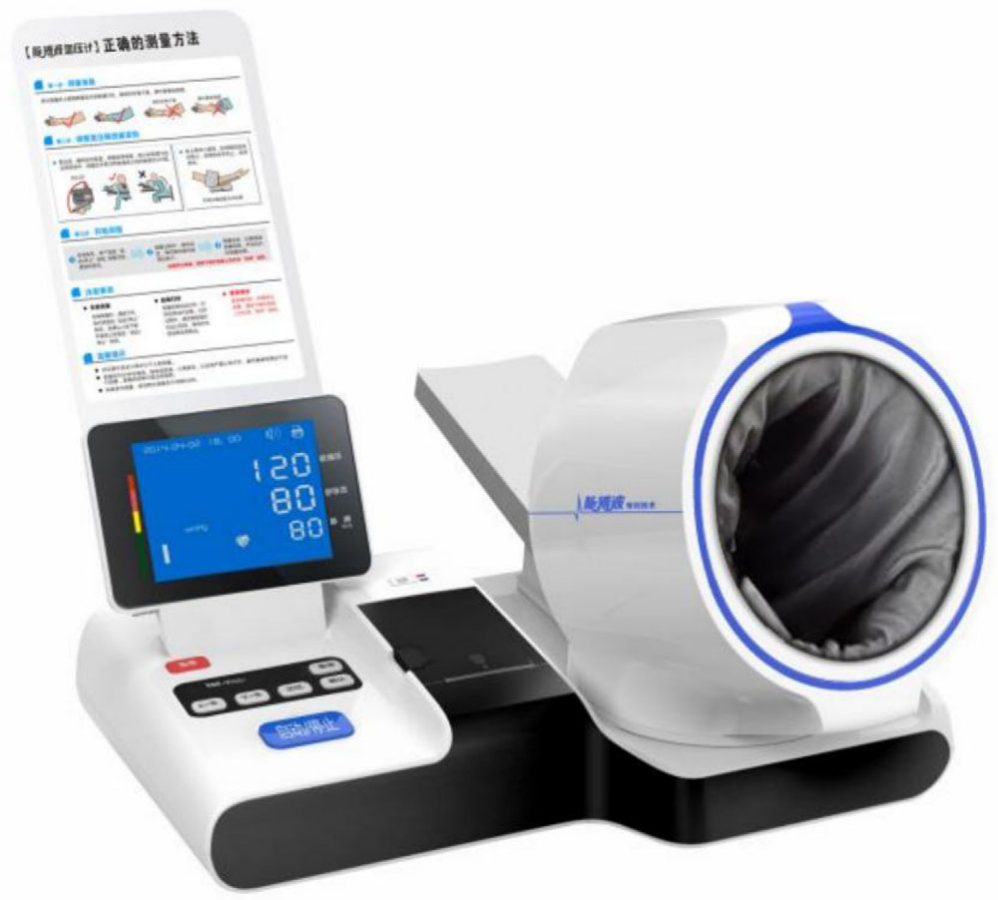
The Raycome model RBP‐9000c.

The Raycome model RBP‐9000c (Figure 1) is an automated, kiosk‐type, single upper‐arm cuff oscillometric BP monitor developed for professional use in the office and home settings, that includes a monitor dock, connecting cable, a standard cuff, and a user guide. The system integrates a main unit with an arm‐in tunnel and a built‐in single cuff, a large display, and a thermal printer; it ships with a power supply and a user guide. Measurements can be performed on either the right or left arm. An intelligent positioning system helps align the elbow/upper arm, and the forearm tunnel can be rotated about ± 10° to facilitate comfortable placement. The device employs a pulse‐wave method with a dual‐airbag and dual‐sensor design that simultaneously captures cuff pressure and pulse‐wave signals; according to the manufacturer, this approach improves waveform acquisition and damping assessment and is claimed to achieve a measurement deviation within ± 2 mmHg. The monitor provides instant voice prompts and result read‐back, automatic printouts, and onboard storage for up to 100 measurement sets. The cuff suits arm circumferences from 17 to 42 cm, and antimicrobial materials are used on contact surfaces for hygiene and ease of cleaning. The specified measurement ranges are: blood pressure 0–300 mmHg (0–40 kPa) and pulse rate 40–180 beats/min. The stated measurement accuracy is ± 2 mmHg (± 0.267 kPa) for blood pressure and ± 2% for pulse rate. Electrical specifications are AC 100–240 V, 50–60 Hz, 1.6–0.8 A. Recommended operating conditions are 5–40°C and 15–80% RH, with transport/storage conditions of −20–55°C and ≤ 93% RH; operating atmospheric pressure 80–106 kPa (transport/storage 50–106 kPa). The unit's external dimensions are approximately 471.5 mm (L) × 402.0 mm (W) × 309.0 mm (H), and the weight is about 8.0 kg. One RBP‐9000 c unit was used in the validation study.

### 2.2 Participants

Regarding the AAMI/ESH/ISO Universal Standard, for a general population validation study of a BP monitor, at least 85 subjects aged > 12 years are required [1, 4]. Table 1 shows the participant selection process. We recruited 105 subjects ≥ 18 years from patients attending the outpatient hypertension clinic (95.3%) and from hospital staff (4.7%). Of these, 20 were excluded. Reasons for exclusions were arrhythmia (*n* = 5), reference BP variability (*n* = 3), sound not audible (*n* = 2), too fragile to attend all the measurements (*n* = 2), and subject talking during BP measurement (*n* = 8). Finally, 85 subjects were analyzed (55.3% male).

**TABLE 1:** Subjects recruited and excluded from the analysis.

	Subjects
Recruited	105
Excluded	20
Reasons for exclusion	
Arrhythmia	5
Reference BP variability (> 12/8 mmHg for systolic/diastolic)	3
K sounds not audible	2
Talking during BP measurements	8
Too fragile	2
Analyzed	85

### 2.3 Validation Team

The study was conducted in a BP measurement research laboratory by a supervisor (Yuqing Zhang) and two trained observers (Shijie Yang and Zhanyang Zhou). Both observers held certificates of Good Clinical Practice (GCP) and were strictly retrained in mercury BP measurement. The supervisor (Yuqing Zhang), who also obtained GCP certification before the research initiation, provided training to the observers on the testing procedures and the operational approach of the test device. Before the main test, a simulation test using a mercury sphygmomanometer was conducted to confirm that the observers could accurately determine the SBP and DBP by correctly listening for the Korotkoff sounds, particularly the fifth phase (K5). Besides, Huanhuan Miao helped with data management (or experimental data collection).

### 2.4 Reference Blood Pressure Measurement

Two connected (Y‐tube) standard mercury sphygmomanometers (XJ11D, Shanghai Medical Instruments Co. Ltd, China), which had been calibrated before the study initiation, were used for simultaneous reference auscultatory BP measurements by two observers using a dual‐head teaching stethoscope (Shanghai Taihuhuamei Medical Instruments Co. Ltd, China). Four cuffs with inflatable bladder dimensions 9 × 18, 12 × 23, 14 × 28, and 16 × 33 cm were used so that the length would cover 75–100% of the individual participant's midarm circumference and the width 37%–50% [1].

### 2.5 Procedure

The arm sequential method was applied according to the recommendations and practical guidance from performing and reporting validation studies [1]. Briefly, we included two entry BP measurements (reference R0 and test device T0) followed by four reference measurements (R1, R2, R3, and R4) taken alternately with three test device measurements (T1, T2, and T3). All measurements were performed on the left arm. The observers were blinded to each other's readings and the test device results. The supervisor (Yuqing Zhang) registered the RBP‐9000 c measurements and checked the observers’ measurements. In case of disagreement between the observers exceeding 4 mmHg, additional pairs of measurements were performed. A maximum of eight pairs of BP determinations was allowed, after which the subject was excluded.

### 2.6 Statistical Analysis

Continuous variables are expressed as the mean ± SD, and categorical variables are expressed as frequencies or percentages. Standardized Bland–Altman scatterplots of the differences between the RBP‐9000 c and the reference BP measurements versus their mean were performed. The AAMI/ESH/ISO Universal Standard (ISO 81060–2:2018) requirements were strictly followed [1]. Data were analyzed with IBM SPSS Statistics version 24 statistical software.

### 2.7 Study Approval

The study protocol was approved by the ethics committee of Fuwai Hospital, Chinese Academy of Medical Sciences and Peking Union Medical College, Beijing, China. All participants signed an informed consent before inclusion in the study, in accordance with the Declaration of Helsinki and WHO standards for observational studies [5]. The confidentiality of the subjects was guaranteed at all times in accordance with the provisions of current legislation on personal data protection (Official Law 15/1999 of December 13 on Protection of Personal Data), and the conditions were contemplated by Act 14/2007 on biomedical research.

## 3 Results

One hundred and five individuals were recruited, and 85 were analyzed. The participants’ characteristics are shown in Table 2. The Universal Standard requirements for age and gender were fulfilled [1]. The mean BP difference between the simultaneous observers’ measurements was 0.3 ± 1.6 mmHg for SBP and 0.4 ± 2.0 mmHg for DBP (range −4 to 4 mmHg for both SBP and DBP). Twelve BP readings with interobserver disagreement > 4 mmHg were recorded.

**TABLE 2:** Participants’ characteristics (*n* = 85).

	Mean ± SD	Range
Age (years)	50.2 ± 14.3	22–75
Gender (male/female)	47/38	
Arm circumference	31.1 ± 5.3	18–42
Entry SBP R0 (mmHg)	140.5 ± 23.9	86–201
Entry DBP R0 (mmHg)	88.1 ± 16.9	42–126

Table 3 shows the distribution of the reference BP measurements R1–R4. The Universal Standard requirements for BP distribution were fulfilled [1]. The validation analysis is shown in Table 4. Both criteria 1 and 2 suggested ‘pass’ for SBP and DBP [1].

**TABLE 3:** Distribution of reference blood pressure measurements (R1–R4).

SBP	≤ 100 mmHg	≥ 160 mmHg	≥ 140 mmHg
	8.3%	18.1%	45.3%
DBP	≤ 60 mmHg	≥ 100 mmHg	≥ 85 mmHg
	5.1%	20.8%	47.9%

Abbreviations: DBP, diastolic blood pressure; SBP, systolic blood pressure.

**TABLE 4:** Validation study results.

		Archieved		
	Pass requirement	SBP		DBP
Criterion 1 (255 BP pairs)				
Mean BP difference (mmHg)	≤ 5	2.4		3.3
SD (mmHg)	≤ 8	6.7		6.3
		Pass		Pass
Criterion 2 (85 Subjects)				
SD (mmHg, SBP/DBP)	≤ 6.51/6.09	5.28		5.32
		Pass		Pass
Result			Pass	

**FIGURE 2:**
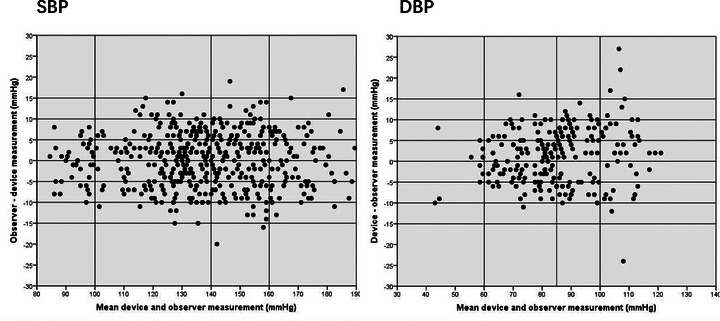
Standardized Bland–Altman scatterplots of the RBP‐9000c—reference BP differences.

The manufacturer provided the test device that supports participants with an arm circumference of 18–42 cm, and the mean arm circumference in the study was 31.1 ± 5.3 cm. The number of participants with large arm circumference (32–42 cm), medium arm (22–32 cm), and small arm (18–22 cm) was 16, 43, and 26, respectively. Regarding the AAMI/ESH/ISO Universal Standard, for each test device cuff, at least 40% of the subjects included using this cuff must have an arm circumference within the upper half of the specified range of use of the cuff, and at least 40% within the lower half [1]. The Universal Standard requirements for BP distribution were fulfilled [1]. Standardized Bland–Altman scatterplots of the RBP‐9000c—reference BP differences against their mean are illustrated in Figure 2.

## 4 Discussion

Blood pressure measurement is the cornerstone for hypertension clinical management, and therefore, its accuracy is of utmost importance. To prevent the methodology itself from being a source of error, it is essential that the quality of the devices be rigorously evaluated, as this point is central to the method's clinical validity. It is accepted that all blood pressure measuring devices must be validated independently in the clinical setting. This study is a typical application of the AAMI/ESH/ISO Universal Standard (ISO 81060–2: 2018) for the validation of the professional office and home recorder BP monitor RBP‐9000c in the general population. As shown by the Bland–Altman plots, only 5 and 5 dots were beyond the 15 mmHg limit for systolic and diastolic blood pressures, respectively. In addition, we recruited a large number of participants with blood pressure exceeding 160/100, including several extremely hypertensive patients, and found that RBP‐9000c could be measured accurately in these cases. Both systolic and diastolic blood pressure readings with RBP‐9000c meet the guidelines. In fact, our study was conducted rigorously in accordance with the guidelines. Therefore, we believe there may not be any significant limitations. There were no issues with the device use or the application of the validation protocol during the study. In conclusion, the analysis showed that the RBP‐9000c device fulfills all the accuracy criteria of the Universal Standard in the general population and can be recommended for clinic and self‐use at home.

## Funding

The authors received no specific funding for this work.

## Ethics Statement

The study protocol was approved by the ethics committee of Fuwai Hospital, Chinese Academy of Medical Sciences and Peking Union Medical College, Beijing, China (2022–ZX051).

## Consent

All participants signed an informed consent before inclusion in the study.

## Conflicts of Interest

The authors declare no conflicts of interest.

## Data Availability

The datasets generated during and analyzed during the current study are not publicly available, but are available from the corresponding author on reasonable request.
